# Single Virus Targeting Multiple Organs: What We Know and Where We Are Heading?

**DOI:** 10.3389/fmed.2020.00370

**Published:** 2020-08-05

**Authors:** Ashish Prasad, Manoj Prasad

**Affiliations:** National Institute of Plant Genome Research, New Delhi, India

**Keywords:** SARS-CoV-2, symptoms, COVID-19, disease symptoms, multiple organ failure, health effects

## Abstract

COVID-19 caused by SARS-CoV-2 has already infected more than 6. 3 million people worldwide as of 1st June 2020 and caused a global medical emergency. Healthcare professionals have been struggling to devise appropriate therapeutic strategies against the virus mainly due to the diverse range of symptoms and multiple-organ failure in infected patients. Several broad-spectrum antiviral drugs are being used for treatment; however, there is yet no specific drug or vaccine against the virus. Multiple-organ failure due to hyperactivity of the immune system resulting in cytokine storms is a major reason for death among the 5% critically ill patients. In this article, we have discussed the damage caused by COVID-19 on different organs of the human body.

## Introduction

Severe acute respiratory syndrome coronavirus 2 (SARS-CoV-2), which is the causal agent of Coronavirus disease 2019 (COVID-19), is a single-stranded RNA virus with a non-segmented genome. Like other coronaviruses, four structural proteins are present in assembled viruses: membrane protein (M), capsid protein (C), envelope protein (E), and spike glycoprotein (S) (1, 2). The receptor-binding domain (RBD) present within the S-glycoprotein is essential for binding to host angiotensin-converting enzyme 2 (ACE2) which serves as a receptor for viral entry. The RBD of SARS-CoV-2 has greater affinity for ACE2 than SARS-CoV (3). The presence of ACE2 throughout the human body marks tissues vulnerable to the virus. The receptor is present in the brain, liver, spleen, kidney, bone marrow, thymus, nasopharynx, oral, and nasal mucosa, lung, small intestine, stomach, colon, lymph nodes, skin, arteries, and veins (4).

**Figure 1:**
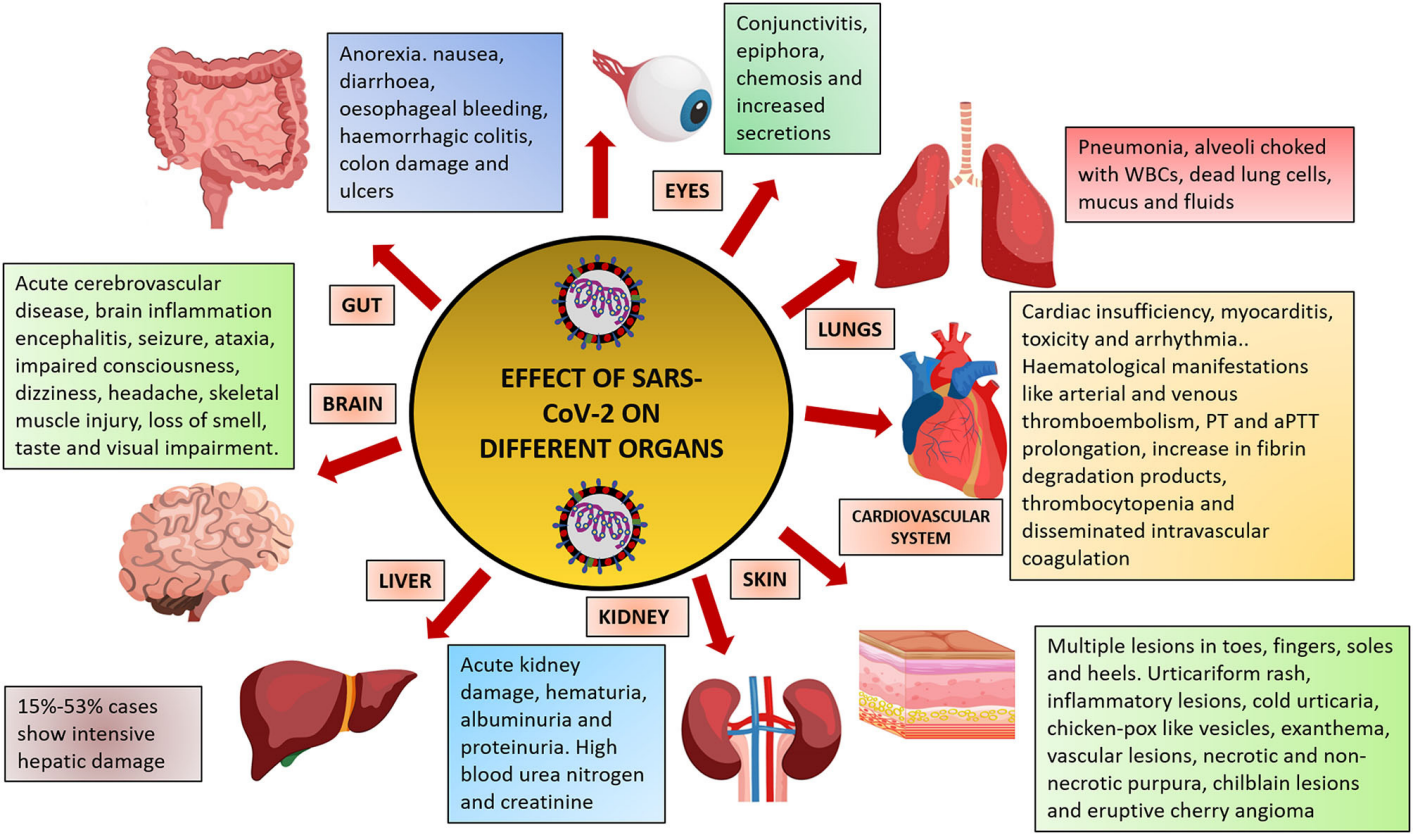
Effect of SARS-CoV-2 infection on different organs of the human body. Figure prepared using images from freepik.com.

Due to the lack of vaccine, immunization against the virus is not yet possible (5). Drugs like remdesivir and chloroquine are currently being used for treatment (6); however, the standard of care and use of drugs is varied between countries. To understand the biology and pathogenesis of the virus, many research papers have been published which are serving as important resources for developing diagnostics. However, the exact nature and extent of damage that the virus can cause is still not well-known. Although most of the patients develop severe respiratory problems, other organs like the brain, blood vessels, heart, gut, liver, and kidney are also susceptible to damage (Figure 1). This article summarizes the complications caused by the virus on different organs of the body.

## Immunological Response To SARS-CoV-2 and Cytokine Storm

Cytokine storm is the flaring-out-of-control inflammatory response of the immune system mediated by excessive production of pro-inflammatory cytokines. The major types of cytokines include interferons, interleukins, chemokines, colony-stimulating factors, and tumor necrosis factor (7). They are involved in the initiation, amplification, and regulation of adaptive immunity (8). The damage-associated molecular patterns are recognized by epithelial cells, endothelial cells, and alveolar macrophages which start releasing cytokines like IL-6 and IP-10. T-cells, monocytes, and macrophages migrate to the site of infection and help in the clearance of infected cells (9). In a study with 20 COVID-19-recovering patients in USA, virus-specific CD4^+^ and CD8^+^ T cells have been identified in 100 and 70% of the convalescent patients, respectively. CD4^+^ T cell response was robust with maximum IgA and IgG against spike and membrane protein of SARS-CoV-2 (10). In another study with COVID-19 patients in China, an early response of IgA instead of IgG was observed in the humoral immune response against SARS-CoV-2 (11). IgA response is also much stronger and persistent than IgM response (11). However, in some patients, a defective immune response results due to the overproduction of pro-inflammatory cytokines leading to cytokine storm (12). A higher level of cytokines leading to inflammation in multiple organs has been observed in intensive care unit (ICU) patients than in non-ICU patients (13). Multiple-organ damage is a result of the cytokine storm circulating to other organs (9).

## Radiological Aspects of COVID-19

Radiological imaging is important for the diagnosis and assessment of disease progression. Just like other pneumonias caused by other coronaviruses, asymmetric, and diffuse airspace opacities have been observed in patients (14). Transverse chest CT-scan images have revealed bilateral ground glass opacities and areas with sub-segmental consolidation (13). Certain other features like round opacities, crazy paving, and reticulation along with peripheral opacities are also common findings in chest CT scans (15). CT is recommended for recovered as well as recovering patients to evaluate long-term lung damages like fibrosis as observed in other coronavirus infections like SARS-CoV and Middle East respiratory syndrome coronavirus (MERS-CoV) (16).

## Effect on The Respiratory System

There is considerable debate on the air-borne transmission of SARS-CoV-2 with no compelling evidence (17). A report suggests that the virus can remain infectious in aerosols for up to 3 h (18). The nasal and throat epithelial cells serve as safe havens for the virus due to the high expression of ACE2 in these cells (19). The virus starts multiplying in these cells and, if not neutralized by the immune system, is shed in large quantity, which leads to spread to other individuals. The next target of the virus are the lungs which are also rich in ACE2 receptors (4). Type-II pneumocytes are involved in the production of alveolar surfactant, which reduces the surface tension of alveoli and airway walls preventing their collapse and allowing O_2_/CO_2_ exchange (20). Virus entry into these cells affects their normal physiology and associated processes, which may lead to lung collapse. In the meantime, the host cells recognize the virus/viral proteins as foreign antigens and lymphocytes start producing immunoglobulin M (IgM), IgA, and IgG (10, 21). It has been observed that 5% of COVID-19 patients become critically ill with severe pneumonia and multiple-organ damage and cytokine storm might be a possible explanation for such an observation. The dead cells and fluid left behind due to the exaggerated immune response choke the alveoli and airways, thus hampering O_2_/CO_2_ exchange and development of pneumonia. Chest x-rays and CT scans reveal white opacities at regions where black space (air) should be present (13). This is due to choking of alveoli with WBCs, fluid, mucus, and dead lung cells (22).

## Effect on Kidneys

Kidneys are another target for SARS-CoV-2 due to the presence of ACE2 receptors in the proximal tubules and glomeruli (23). Proteinuria, hematuria, and elevated blood urea nitrogen and creatinine are a common observation in COVID-19 patients (24, 25). Plasma proteins are absorbed in the proximal tubules, and injury to these tubules results in increased protein excretion in urine (26). Hematuria can be caused by glomerular barrier disruption (27). However, proteinuria and hematuria have been described as general findings and further studies are required to shed light into their characteristics. SARS-CoV-2 RNA has been detected in urine samples by RT-PCR, and fully assembled viruses have been observed by transmission electron microscopy (28). Transmission by urine is another putative source of spread and should be considered while devising management strategies. Electron microscopy has also revealed the presence of viral particles in proximal tubules (29). In a renal histopathological study of 26 deceased patients in Tongji Medical College, China, revealed acute proximal tubule damage along with clusters of SARS-CoV-2 in podocytes and tubular epithelium (30). The systemic immune response, cytokine storm, direct viral infection, and other hemodynamic factors in critically ill patients might be responsible for AKI leading to death of many patients due to kidney failure.

## Effect on Heart and Blood Vessels

Heart and blood vessels are the other hotspots of the ACE2 receptor, thus being prone to SARS-CoV-2 (4). Out of 416 patients from Renmin Hospital in Wuhan, 19.7% suffered from severe heart injuries, highlighting the risk of heart failure associated with the infection (31). Another report suggested arrhythmia in 16 out of 36 ICU patients in Zhongnan Hospital in Wuhan (32). Patients in the hospital were receiving antiviral therapy (precise drug not specified), and antiviral drugs like hydroxychloroquine are known to promote arrhythmias (33). Myocarditis caused by inflammation of the myocardium has also been observed (34). Antiviral drugs have a very high risk potential for the heart and may lead to cardiac insufficiency, toxicity, and arrhythmia (35). Ribavirin, which is being used for treatment (36), is reported to induce sick sinus syndrome (37). Thus, it is important that the cardiovascular system is closely monitored in COVID-19 patients and special attention must be provided for its protection. A study on 184 infected patients revealed that 31% had thrombotic complications, which was very high, and arterial and venous thromboembolism due to excessive inflammation was a high risk factor (38). The major risk associated with thromboembolism is that blood clots may reach the lungs, brain, and heart and cause pulmonary embolism, stroke, and cardiac arrest, respectively. Kawasaki disease has been reported among children aged 4–11 years who were also infected with SARS-CoV-2 (39). A 6-months-old COVID-19 patient developed bulbar conjunctival infection, erythema, and edema in the upper extremities along with maculopapular rash and persistent fever. The respiratory symptoms like cough, congestion, or rhinorrhea were normal (40). Another case of a 5-years-old represented the development of incomplete Kawasaki disease (41). It is necessary to investigate the clinical course of pediatric patients with COVID-19 in association with Kawasaki disease.

## Effect on The Gastrointestinal Tract

A report suggests that out of 95 patients positive for COVID-19 from Sun Yat-sen University Hospital in China, 58 cases had gastrointestinal (GI) symptoms (42). Anorexia, Diarrhea, and nausea were the main symptoms among the patients, and esophageal bleeding and ulcers were reported from a patient with severe illness. However, the relation of ulcers and esophageal bleeding with SARS-CoV-2 infection is not yet clear and is just an observational study. Stomach, esophagus, duodenum, and rectum tissues have been found to be positive for the virus, indicating that these organs could also act as possible direct targets for transmission (42). ACE2 receptors are highly expressed in the small intestine, colon, and stomach, making the gastrointestinal tract highly susceptible to SARS-CoV-2 infection (4). Endoscopy has revealed extensive colon injury with hemorrhagic colitis, and stool has tested positive for viral RNA in some patients (43, 44). However, there is still no evidence that viral transmission can occur through stool. Healthy gut microbiota is important for proper functioning of the gut. Microbial dysbiosis has been observed in COVID-19 patients with a maximum effect on *Bifidobacterium sp*. and *Lactobacillus sp*. (45). It will be interesting to see whether probiotic bacteria can be used as therapeutics for controlling GI symptoms caused by the virus. However, we do not know anything about the probiotic use against the virus and studies in this area must be conducted to gain further insights.

## Effect on The Central and Peripheral Nervous Systems

A case study on 214 COVID-19 patients from three special care centers of Union Hospital, Wuhan, revealed that 36.4% of the infected people had neurologic symptoms (46). The symptoms were very diverse and ranged from acute cerebrovascular disease, to seizure, to ataxia, to impaired consciousness, to dizziness, to headache, and to skeletal muscle injury. Loss of smell and taste and visual impairment were also the effects caused by the virus on the peripheral nervous system (46). The olfactory epithelium lies in the nasal cavity and is a site for viral replication and possible entry site to the brain (47). The central and peripheral nervous systems are also very rich in ACE2 abundance, making them susceptible to SARS-CoV-2 infection (4). Although shortness of breath due to lack of oxygen (silent hypoxemia) is one of the symptoms of the disease, in some cases, no such symptom has been observed although blood oxygen is critically low. Brainstem reflex is responsible for the sensing of oxygen starvation (48), and its decrease might be a possible explanation for such a manifestation. Silent hypoxemia is a difficult manifestation to control because patients might appear normal but with conditions deteriorating suddenly at a rapid pace (49). Measuring the peripheral oxygen level is very important for any COVID-19 patient, as several reports suggest patients with minimal symptoms but with very critical oxygen level (49, 50). Brainstem dysfunction also results in altered sleep–wake cycles and compromised autonomous control of the immune, circulatory, digestive, and respiratory systems (51). Brain inflammation encephalitis accompanied with seizures has also been reported (52). SARS-CoV-2 has been detected in cerebrospinal fluid in some cases, suggesting that the neurological manifestations might be a result of the direct invasion of CNS and PNS by the virus (52). However, more studies and autopsies are required to associate the nervous system manifestations with direct viral invasion.

## Effect on The Liver

Abnormal alanine transaminase (ALT) and aspartate aminotransferase (AST) levels are an indication of liver damage. Liver damage has been observed in about 15–53% of the cases with elevated bilirubin, AST, and ALT (53). Mild lobular and portal damage and microvascular steatosis have been observed in liver biopsies of COVID-19 deceased patients (22). The adverse effects of antiviral drugs like hydroxychloroquine, which is reported to cause acute toxic hepatitis (54) and cytokine burst, might be responsible for such high percentage of hepatic damage cases in severely ill patients.

## Effect on The Eyes

The occurrence of conjunctivitis has also been reported as a clinical manifestation of the infection and tears, and conjunctival fluids have tested positive for the virus in such cases (55). A doctor in China who tested positive for the virus had developed conjunctivitis before the appearance of respiratory symptoms, and it is believed that he was infected through ocular fluids (56). Some other ocular manifestations that have been observed are epiphora, chemosis, increased secretions, and conjunctival hyperemia (57). A study conducted on 56 patients in Wuhan revealed that aggravated ocular symptoms had occurred in 27% of the patients just before the onset of respiratory symptoms (58). This suggests that ocular manifestations are relatively common in COVID-19 patients and should also be considered during diagnosis (59). Long-term usage of antiviral drugs like chloroquine and hydroxychloroquine is a concern because it is known to cause retinal toxicity and retinopathy (60).

## Effect on The Skin

Some clinical manifestations have also been observed in the skin, hands, and toes 3–4 weeks after COVID-19 infection. In a study, out of six Spanish patients that had similar manifestations, two were tested positive for the virus weeks before. Multiple lesions were observed in the toes, fingers, soles, and heel (61). In another study, a 32-years-old woman from Ramon y Cajal Hospital, Madrid, Spain, developed urticariform rash 6 days after COVID-19 symptom onset. She was treated with hydroxychloroquine and azithromycin. Some other patients developed maculopapular rash (62). A nationwide study on skin manifestations across France reported several skin manifestations in COVID-19 patients. The manifestations ranged from inflammatory lesions, cold urticaria, chicken pox-like vesicles, exanthema, vascular lesions, necrotic, and non-necrotic purpura, chilblain lesions, and eruptive cherry angioma (63). There are several other reports suggesting skin manifestations in COVID-19 patients (64, 65). However, these are just observational reports and more studies need to be conducted to associate these observations with SARS-CoV-2 infection.

## Conclusion

According to World Health Organization, the common symptoms of the disease include dry cough, fatigue, and fever. Sore throat, nasal congestion, body aches, and pains are also common. However, several other manifestations related to the heart, lungs, liver, kidney, central nervous system, and gastrointestinal system failure is making treatment difficult for critically ill patients (66). It is still unclear whether it is the virus itself or the exaggerated immune response of the body that is causing critical illness in about 5% of the infected patients. Several clinical trials are underway, aiming at immunosuppression of COVID-19 patients to reduce cytokine storm-related manifestations resulting in multiple-organ damage (9). However, this reduces the natural capability of the body to fight against the virus. Antiviral drugs such as remdesivir, ritonavir, and lopinavir are also being used; however, they are known to cause severe side effects on different organs (6). The presence of ACE2 receptors throughout the human body (4) provides an opportunity for the virus to invade different organs easily and is a possible explanation why multiple-organ damage has been reported in many patients. Knowledge on the potential organ injuries associated with COVID-19 will help in the implementation of protective measures and proper management of the disease.

## Author Contributions

MP conceived the idea. AP wrote the manuscript and provided revisions to the manuscript. All authors have read and approved the final version of the manuscript.

## Conflict of Interest

The authors declare that the research was conducted in the absence of any commercial or financial relationships that could be construed as a potential conflict of interest.
